# Anxiolytic-Like Actions of Fatty Acids Identified in Human Amniotic Fluid

**DOI:** 10.1155/2013/823289

**Published:** 2013-04-30

**Authors:** Rosa Isela García-Ríos, Juan Francisco Rodríguez-Landa, Carlos M. Contreras

**Affiliations:** ^1^Laboratorio de Neurofarmacología, Instituto de Neuroetología, Universidad Veracruzana, Avenue Dr. Luis Castelazo s/n Col. Industrial Las Ánimas, 91190 Xalapa, VER, Mexico; ^2^Unidad Periférica Xalapa del Instituto de Investigaciones Biomédicas (UNAM), Xalapa, VER, Mexico

## Abstract

Eight fatty acids (C12–C18) were previously identified in human amniotic fluid, colostrum, and milk in similar proportions but different amounts. Amniotic fluid is well known to be the natural environment for development in mammals. Interestingly, amniotic fluid and an artificial mixture of fatty acids contained in amniotic fluid produce similar anxiolytic-like actions in Wistar rats. We explored whether the lowest amount of fatty acids contained in amniotic fluid with respect to colostrum and milk produces such anxiolytic-like effects. Although a trend toward a dose-response effect was observed, only an amount of fatty acids that was similar to amniotic fluid fully mimicked the effect of diazepam (2 mg/kg, i.p.) in the defensive burying test, an action devoid of effects on locomotor activity and motor coordination. Our results confirm that the amount of fatty acids contained in amniotic fluid is sufficient to produce anxiolytic-like effects, suggesting similar actions during intrauterine development.

## 1. Introduction

Fatty acids (FATs) are organic lipid compounds that consist of a hydrocarbon chain of variable length and carboxyl group at the end of the chain [1]. The metabolic processes of FATs guarantee their content in the organism [2–4], suggesting other functions besides their nutritional role. Fatty acids are structural components of cellular membranes and participate in the modulation of ion channels [5], at least in myocardial cells and neurons [6, 7]. During embryonic development, FATs play an important role in development. Some long-chain polyunsaturated FATs are structural components of neuronal membranes from the second half of gestation [8]. Dietary supplementation with long-chain FATs during gestation and childhood improves visual function and cerebral processes related to language [9, 10]. In contrast, an early FAT restriction diet enhances the vulnerability to stress during adulthood in humans and laboratory rats [11–13].

We recently reported that human amniotic fluid, colostrum, and milk consistently contained eight FATs (C12:0, lauric acid; C14:0, myristic acid; C16:0, palmitic acid; C16:1, palmitoleic acid; C18:0, stearic acid; C18:1 *cis*, oleic acid; C18:1 *trans*, elaidic acid; C18:2, linoleic acid) in similar proportions. The highest amounts were detected in maternal milk, and the lowest amounts were detected in amniotic fluid. An artificial mixture of these FATs acts as a sensorial cue that guides newborns to the maternal breast [14], supporting the observation that some sensorial cues modulate emotional states in receptor subjects [15, 16]. 

Earlier observations indicated that olfactory stimulation with maternal amniotic fluid or maternal milk decreased grimaces and crying in human newborns when their mothers were absent [17, 18]. An equivalent calming effect was observed in several mammalian species, including rabbits, pigs [19], rats [20], sheep [21], and goats [22], seemingly aiding the transition from intrauterine life to the postnatal environment [23]. Additionally, both amniotic fluid and an artificial mixture of its FATs produced anxiolytic-like effects similar to diazepam in adult rats in the defensive burying test and elevated plus maze [24].

Because amniotic fluid may exert multiple functions [25] besides providing a regulated temperature during development [26], we hypothesized that an artificial mixture that contains a similar amount of FATs as amniotic fluid, but not less, may produce anxiolytic-like actions in experimental models. Consequently, the present study evaluated the anxiolytic-like effects of artificial mixtures that contained four different amounts of FATs in Wistar rats subjected to the defensive burying test [27]. Additionally, the open field [28] and rotarod [29] tests were used to discard any possible influence of changes in spontaneous locomotor activity on the anxiety test.

## 2. Methods

### 2.1. Ethics

All of the experiments were performed in accordance with international ethical standards based on the Guide for the Care and Use of Laboratory Animals [30]. The general protocol received authorization from the Biomedical Research Institute Ethical Committee (Universidad Nacional Autónoma de México).

### 2.2. Subjects

Seventy-one adult male Wistar rats were obtained from a local strain initially supplied by Harlan (Mexico City, Mexico). The rats were housed in local housing facilities at a mean temperature of 25 ± 2°C with a 12 h/12 h light/dark cycle (lights on at 7:00 AM). The rats included in the study were approximately 3-month old, weighed 250–300 g, and were housed five to six per cage in acrylic boxes (44 cm width × 33 cm length × 20 cm height) with *ad libitum* access to food (Teklad lab animal diets; Harlan) and purified water. All of the experiments were performed during the light period (approximately 12:00 PM).

### 2.3. Preparation of Artificial Mixture of Fatty Acids

A mixture of eight FATs was prepared according to previous studies [14]. The artificial mixture of FATs was prepared in 100 mL of vehicle (96% propylene glycol and 4% ethanol) at a temperature <40°C. Each milliliter contained lauric acid (4.0 *μ*g), myristic acid (30.0 *μ*g), palmitic acid (153.0 *μ*g), palmitoleic acid (71.0 *μ*g), stearic acid (37.0 *μ*g), oleic acid (80.0 *μ*g), elaidic acid (15.0 *μ*g), and linoleic acid (44.0 *μ*g). All of the chemical compounds were analytical grade and obtained from Sigma-Aldrich (St. Louis, MO, USA).

### 2.4. Behavioral Tests

#### 2.4.1. Defensive Burying Test

An acrylic box (27 × 17.5 × 15.5 cm) with the floor covered by a 5 cm bed of fine sawdust (Teklad Sani-Chips 7090, 2.2 cubic feet; Harlan, Indianapolis, IN, USA) was placed inside a noise-isolated box (65 × 55 × 45 cm; Coulbourn Instruments, Whitehall, PA, USA). An electrode (7 cm length, 0.5 cm diameter) protruded 2 cm above the sawdust bed horizontally from one wall of the box (17.7 × 15.5 cm). The electrode delivered a constant-intensity direct current (0.3 mA) from an electronic stimulator (Grass Instruments S44, Quincy, MA, USA) coupled in series to a stimulus isolation unit (Grass Instruments SIU5) and constant-current unit (Grass 7 Instruments CCUIA). When a rat incidentally touched the electrode, it received an electric shock and began to vigorously displace the sawdust to cover the electrode. All of the sessions were recorded for subsequent analysis by two independent observers to measure the burying latency and cumulative burying time during a 10 min test. After each test session, the bed of fine sawdust was removed and replaced by clean sawdust bedding. Only observations with more than 95% agreement between observers were included in the data analysis. Immediately after the defensive burying test, each rat was evaluated in the open field test.

#### 2.4.2. Open Field Test

To evaluate the effects of the treatments on spontaneous locomotor activity, the rats were subjected to a 5 min open field test. We used an automated motor activity monitor (Acti-Track v2.7.10, PanLab, S.L. Instrument, Barcelona, Spain) in a perspex box (45 × 45 cm base, 35 cm height). A total of 32 infrared beams, 16 each on perpendicular walls, were mounted 3 cm above the box frame floor and connected to an interface (LE 8811, LSI Letica Scientific Instruments, Barcelona, Spain). The data were sent to a computer. For data analysis, the floor of the cage was divided into five equally sized virtual squares (four peripheral squares and one central square), and we measured the total number of entries into the squares (i.e., crossings), time spent active (in seconds), and time spent resting (in seconds).

After each experimental session, the open field box was carefully cleaned and deodorized with a cleaning solution (0.5% ammonia, 15% ethanol, 10% extran, 5% isopropyl alcohol, 19% Pinol, and 50.5% water). Five minutes elapsed between each test to allow the scent of the substances to dissipate.

#### 2.4.3. Rotarod Test

To assess the effects of the treatments on motor coordination, all of the rats underwent a 3-day training program on a 7-cm diameter rotarod (LE 8300, LSI Letica, Panlab Scientific Instruments, Barcelona, Spain). During the training period, each rat was placed on a horizontal rod that rotated at a gradually increasing speed of 4 to 20 rotations per minute for a maximum period of 5 min to determine baseline performance. The day after training, motor coordination was recorded by gradually increasing the speed from 4 to 20 rotations per minute during five trials with a cutoff time of 3 min. The dependent variable was the total time spent on the rotating rod (in seconds).

### 2.5. Anxiety-Like Behavior in Defensive Burying Test

 We used a transversal design, with six independent groups. One group received vehicle (1.0 mL/rat, s.c.; *n* = 8), and the other four groups received (s.c.) different volumes of the artificial FAT mixture (1 mL, *n* = 9; 0.5 mL, *n* = 6; 0.25 mL, *n* = 6; 0.125 mL, *n* = 6) in such a manner that the rats that received 1, 0.5, 0.25, and 0.125 mL received a similar proportion of FATs but one-half, one-quarter, and one-eighth of the total amount of FATs contained in amniotic fluid (Table 1). The volume administered in each treatment was adjusted to 1 mL/rat by the addition of vehicle. The last group (*n* = 10) received 2.0 mg/kg diazepam (i.p.; Hoffman-Roche, Basel, Switzerland) dissolved in 40% propylene glycol and injected in a volume of 2.0 mL/kg as a reference anxiolytic drug. All of the injections were administered 1 h before the defensive burying test and open field test.

### 2.6. Motor Coordination in Rotarod Test

 After the anxiety test, other groups of rats were subjected to a transversal design, with three independent groups. One group received vehicle (1.0 mL/rat, s.c.; *n* = 8). One group received (s.c.) the artificial FAT mixture (1 mL, *n* = 9), and one group received diazepam (2 mg/kg, i.p.). All of the injections were administered after the training sessions, 1 h before the rotarod test.

### 2.7. Statistical Analysis

The data were analyzed using one-way analysis of variance (ANOVA) for independent groups. Values of *P* ≤ 0.05 in the ANOVA were followed by the Student-Newman-Keuls (SNK) *post hoc* test. The results are expressed as the mean ± SE of each variable evaluated. For graphical representation, the burying latencies and cumulative burying times were fitted to a nonlinear logarithmic curve using Kaleidagraph software (Synergy software).

## 3. Results

### 3.1. Defensive Burying Test

**Figure 1:**
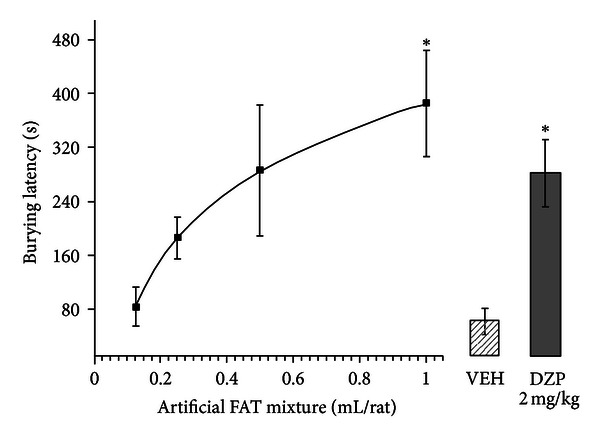
Burying latency. A volume of 1 mL of the artificial FAT mixture and diazepam (2.0 mg/kg) significantly (*P < 0.002, SNK *post hoc* test) increased burying latency compared with vehicle and the 0.125 and 0.25 mL artificial FAT mixture groups. The data points of the dose-response curve were fit to a logarithmic curve (*R* = 0.965). FAT, fatty acid; VEH, vehicle of fatty acid mixture; DZP, diazepam.

The burying latency analysis revealed significant differences among treatments (*F*_5,39_ = 4.695, *P* < 0.002). Although a trend toward a dose-response effect was detected, the *post hoc* test showed that only the groups treated with 1 mL of the artificial FAT mixture and diazepam displayed a significantly longer burying latency than the vehicle group (*P* < 0.05; Figure 1). 

**Figure 2:**
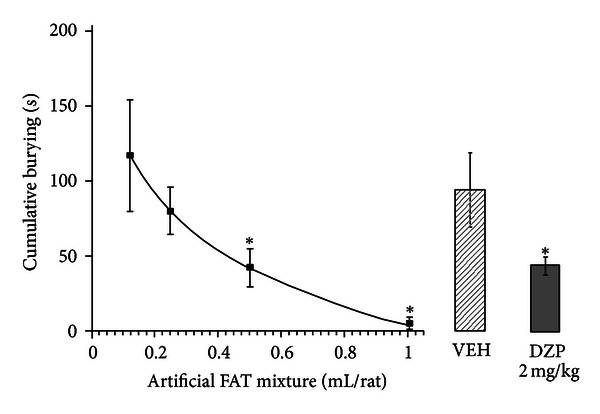
Cumulative burying. Cumulative burying time was significantly less (*P < 0.001, SNK *post hoc* test) in the groups treated with 0.5 and 1 mL of the artificial FAT mixture and diazepam than in the groups treated with vehicle and the other artificial FAT mixture volumes. The data points of the dose-response curve were fit to a logarithmic curve (*R* = 0.9674). FAT, fatty acid; VEH, vehicle of fatty acid mixture; DZP, diazepam.

The analysis of cumulative burying time also revealed significant differences among treatments (*F*_5,39_ = 5.792, *P* < 0.001). A trend toward a dose-response effect was detected, but the *post hoc* test showed that only the groups treated with 0.5 or 1 mL of the artificial FAT mixture and diazepam displayed significantly shorter cumulative burying time than the vehicle group (*P* < 0.05; Figure 2) and the groups treated with other volumes of the artificial FAT mixture.

### 3.2. Open Field Test

The analysis of locomotor activity did not detect significant differences between peripheral and central activity in any of the measures. Therefore, we analyzed the total values of these variables during the 5 min test (Table 2). The one-way ANOVA did not reveal significant effects of treatment on activity time (*F*_5,39_ = 1.673, *P* = 0.164), resting time (*F*_5,39_ = 1.687, *P* = 0.161), or the number of crossings (*F*_5,39_ = 1.356, *P* = 0.262).

### 3.3. Rotarod Test

The analysis of the total time on the rotarod did not detect significant differences between the groups treated vehicle (118.9 ± 17.94 s), the artificial FAT mixture (132.1 ± 11.45 s), and diazepam (140.8 ± 16.21 s; *F*_2,24_ = 0.510, *P* = 0.607).

## 4. Discussion

The aim of the present study was to determine whether the amount of FATs contained in amniotic fluid produces anxiolytic-like effects on rats in the defensive burying test. The results demonstrated that only the same amount of FATs found in amniotic fluid, but not lesser amounts, mimicked the anxiolytic-like effects of diazepam, without altering spontaneous locomotor activity or motor coordination.

Other studies demonstrated the anxiolytic-like effects of FATs in humans [31, 32], farm animals [33], and experimental animals [34]. However, these studies seemingly selected the concentrations of FATs arbitrarily, did not use an active anxiolytic drug for comparison, did not explore the presence or absence of dose-response effects, and tested other FATs. The present study based the amount of FATs included in the artificial mixture on the amount found in human amniotic fluid. We also compared the results with diazepam. Although we observed some dose-response effects, only the artificial FAT mixture that contained a similar amount of FATs as amniotic fluid [14, 24] produced actions similar to diazepam in a test widely used to measure anxiolytic effects.

In the defensive burying test, the time that elapsed between the first shock and first attempt at burying (i.e., burying latency) is inversely related to the rat's reactivity [35, 36]. Diazepam and other clinically effective anxiolytics increase burying latency [27]. In the present study, the burying latency was similarly increased by the artificial FAT mixture and diazepam, suggesting some similarities in their mechanisms of action, probably including actions at *γ*-aminobutyric acid-A GABA_A_ receptors. In fact, some FATs modulate the opening of sodium [6], potassium [37], and calcium [38] ion channels. To explain the present results, some studies found inhibitory actions of FATs on chloride channels [39] and increased affinity of GABAergic compounds, such as muscimol and anxiolytic drugs like diazepam, produced by FATs by acting on this receptor [40, 41]. These results suggest that the effects of FATs seemingly involve interactions with GABA_A_ receptors. However, to draw more definitive conclusions, specific studies are needed to elucidate the effects of FATs on GABA_A_ receptors.

Cumulative burying time in the defensive burying test is considered an index of reactive anxiety produced by electric shock. Longer cumulative burying times purportedly reflect greater levels of anxiety [35, 36], whereas cumulative burying time is reduced by diazepam [27] and selective serotonin reuptake inhibitors with anxiolytic-like properties [42]. In the present study, 0.5 and 1 mL of the artificial FAT mixture significantly reduced cumulative burying time, confirming the anxiolytic-like effect of FATs [24]. The novelty of the present results, however, is derived from the finding that the anxiolytic-like actions of FATs, similar to diazepam, occurred at the same concentration and proportion of FATs present in human amniotic fluid [14].

We found a statistically significant dose-response relationship [43]. However, the effects that were comparable to diazepam were observed only with the highest volume tested that contained similar amounts of FATs as human amniotic fluid, illustrating the potency of such a concentration of FATs in producing anxiolytic actions and importance of using a positive control in this type of study. However, one limitation of the present study is that the anxiolytic-like effect was found with a mixture of eight FATs, indicating the need for additional studies that determine whether only one or some of these eight FATs are responsible for the anxiolytic-like effects, including the possibility of interactions between some of the FATs.

Changes in locomotor activity may interfere with performance in the defensive burying test. Therefore, the measure of locomotor activity is commonly used to exclude putative nonspecific drug effects in the defensive burying test [36]. In the present study, the open field and rotarod test results complemented the defensive burying test results, discarding possible changes in alertness or motor coordination that could interfere with burying behavior. None of the treatments produced any change in the open field or rotarod test. Importantly, FATs constitute an important caloric source, and its injection may constitute an additional amount of energy source to a normal diet; however, we did not observe any changes in locomotion, discarding this possibility.

Other behavioral studies on the anxiolytic-like effects of FATs did not include active controls in humans [32, 33], farm animals, or experimental animals [33, 34]. The present study demonstrated that FATs in a similar concentration as that detected in human amniotic fluid produce anxiolytic-like effects that are comparable to diazepam. Therefore, we conclude that an artificial mixture of FATs contained in human amniotic fluid produced anxiolytic-like effects in an experimental model of anxiety at a similar concentration as that found in human amniotic fluid. Lower concentrations of the artificial FAT mixture did not exert significant effects. These data suggest an additional protective effect of amniotic fluid during the intrauterine development of the fetus on its emotional state and confirm previous studies that demonstrated the anxiolytic-like effects of the FAT mixture.

## Figures and Tables

**Table 1:** Fatty acid concentrations contained in volume administered to each rat. The fatty acid concentrations contained in 1 mL of the artificial FAT mixture correspond to the proportion found in human amniotic fluid [14, 24].

Fatty acid	Volume				
	0.125 mL	0.25 mL	0.5 mL	1 mL	Content (*μ*g%)
C12:0 (Lauric)	0.5	1.0	2.0	4.0	0.9
C14:0 (Myristic)	3.7	7.5	15.0	30.0	6.9
C16:0 (Palmitic)	19.1	38.2	76.5	153.0	35.3
C16:1 (Palmitoleic)	8.8	17.7	35.5	71.0	16.4
C18:0 (Stearic)	4.6	9.2	18.5	37.0	8.5
C18:1 *cis* (Oleic)	10.0	20.0	40.0	80.0	18.4
C18:1 *trans* (Elaidic)	1.8	3.7	7.5	15.0	3.5
C18:2 (Linoleic)	5.5	11.0	22.0	44.0	10.1

Total	54 (*μ*g/mL)	108.3 (*μ*g/mL)	217 (*μ*g/mL)	434 (*μ*g/mL)	100

**Table 2:** Effect of treatment in open field test. The data are expressed as mean ± standard error of the mean. No significant differences in activity time, resting time, or crossings were found between groups.

Group	Time active (s)	Time resting (s)	Crossings (*n*)
Vehicle (1 mL)	164.8 ± 7.85	135.2 ± 7.85	98.4 ± 13.69
Artificial fatty acid mixture			
0.125 mL	149.6 ± 14.05	150.4 ± 14.05	92.8 ± 16.01
0.25 mL	133.2 ± 8.34	166.8 ± 8.34	74.0 ± 10.18
0.5 mL	160.5 ± 13.80	139.5 ± 13.80	86.8 ± 16.09
1 mL	152.5 ± 14.69	147.5 ± 14.69	85.0 ± 12.14
Diazepam (2 mg/kg)	117.8 ± 18.15	182.2 ± 18.15	58.2 ± 11.85
